# Modified technique of realtime ultrasound guided percutaneous balloon dilatational tracheostomy through laryngeal mask airway insertion

**DOI:** 10.1186/2036-7902-6-S1-A5

**Published:** 2014-01-31

**Authors:** R Marktanner, A Shafei, M Mostafa, H Hon, Y Zaghloul, A Taha

**Affiliations:** 1 Department of Critical Care Medicine, Sheikh Khalifa Medical City managed by Cleveland Clinic, Abu Dhabi, United Arab Emirates; 2 Department of Anaesthesiology, Sheikh Khalifa Medical City managed by Cleveland Clinic, Abu Dhabi, United Arab Emirates

## Background

The technique of bedside percutaneous tracheostomy (BPT) has become a routine procedure in the ICU. As a common standard, a bronchoscope is used during the BPT in order to assess the upper airway anatomy and to assist guiding the needle while penetrating the tracheal inter-ring space into the correct central positioning. However, using the bronchoscope during the puncture of the trachea holds a potential patient safety risk due to the possibility of inaccurate device placement and associated temporary hypoventilation. Moreover, an accidental needle puncture of the bronchoscope is a frequent problem and leads to high repair costs as well as to prolonged downtime periods of the damaged device. It is therefore important to further improve the BPT technique aiming towards minimizing patient safety risks as well as eliminating avoidable technical and financial burdens for healthcare institutions.

## Objective

We have recently reported a series of 25 patients subjected to BPT combining realtime ultrasound guidance with a balloon dilatational device [1]. The technique of balloon dilatation allows radial outward dilatation and thereby minimizes the bleeding and injury risk of anatomical structures such as tracheal cartilages or the posterior wall of the trachea. Our ultrasound guided series demonstrated accuracy, feasibility and safeness and at the same time eliminated the risk of accidental bronchoscope damage. Using this technique, it was also possible to safely perform a targeted central placement of the tracheostoma into the correct inter-ring space without complications. It was noted however, that properly adjusting the endotracheal tube (ETT) in order to easily penetrate the targeted puncture site was often time consuming - especially in patients with obesity and difficult neck anatomy. In order to further optimize our realtime ultrasound guided BPT protocol, we tested the hypothesis, whether exchanging the ETT with a laryngeal mask airway (LMA) prior to the procedure can further advance this technique without compromising already established patient safety features. As suggested by recent publications, the approach of using a LMA during BPT can provide an easier access to the tracheal puncture site especially in patients with obesity and a difficult neck anatomy [2,3]. Likewise, the risk of ETT cuff puncture or ETT transfixion by the needle or guide wire will be eliminated.

## Patients and methods

In order to compare our own previously reported group (n=25, ETT group) of realtime ultrasound guided BPT procedures with a conventional ETT in place, we changed the protocol to preprocedural LMA ventilation in another group of critically ill patients (n=12, LMA group) scheduled for tracheostomy.

## Results

As control data, we used our own ETT group (15 males and 10 females, mean age 61 years ranging 23 to 102 years), who underwent BPT with ultrasound guidance and applying a balloon dilatational technique. The average time of the procedure in the ETT group was 15.9 minutes and no complications have occured. In the LMA group (9 males and 3 females, mean age 55 years ranging from 29 to 72 years), the same procedural approach was used with the exception of inserting a LMA before the procedure. In the LMA group, the average time of the procedure was 12.4 minutes (p=0.004) and likewise, no complications have occurred.

## Conclusion

We have demonstrated in a previous serious of patients, that combining realtime ultrasound guidance and balloon dilatational technique in performing BPT is easily feasible and safe. By inserting a LMA before the BPT – instead of keeping the ETT in place - we successfully further improved this concept by adding additional beneficial features such as procedural time reduction and improved ultrasound imaging of sublaryngeal anatomical structures. These additional advantages were achieved without compromising the already established patient safety features of the previous technique. In our practise, this technique has become the standard BPT strategy due to its combination of excellent features, e.g. avoidance of intraprocedural tube misplacement and hypoventilation, improved assessment of sublaryngeal anatomical structures, elimination of accidental bronchoscope damage and minimization of procedural time even in technically challenging patients with obesity and difficult neck anatomy.

In our opinion, the strategy of combining realtime ultrasound guided identification of correct tracheal structures, preprocedural insertion of a LMA and the balloon dilatational approach represents the least invasive technique of performing BPT. It can be done easily, safely and significantly further shortened the procedural time.
